# Efficacy and Safety of Low-Dose Atropine on Myopia Prevention in Premyopic Children: Systematic Review and Meta-Analysis

**DOI:** 10.3390/jcm13051506

**Published:** 2024-03-05

**Authors:** Ssu-Hsien Lee, Bor-Yuan Tseng, Jen-Hung Wang, Cheng-Jen Chiu

**Affiliations:** 1 School of Medicine, Tzu Chi University, Hualien 970, Taiwan; hl2200543@tzuchi.com.tw (S.-H.L.); hl2200537@tzuchi.com.tw (B.-Y.T.); 2 Department of Medical Research, Buddhist Tzu Chi General Hospital, Hualien 970, Taiwan; paulwang@tzuchi.com.tw; 3 Department of Ophthalmology and Visual Science, Tzu Chi University, Hualien 970, Taiwan; 4 Department of Ophthalmology, Hualien Tzu Chi Hospital, the Buddhist Tzu Chi Medical Foundation, Hualien 970, Taiwan

**Keywords:** myopia, myopia control, atropine, premyopia, systematic review, meta-analysis, efficacy, safety

## Abstract

**Background:** Early-onset myopia increases the risk of irreversible high myopia. **Methods:** This study systematically evaluated the efficacy and safety of low-dose atropine for myopia control in children with premyopia through meta-analysis using random-effects models. Effect sizes were calculated using risk ratios (RRs) with 95% confidence intervals (CIs). Comprehensive searches of PubMed, EMBASE, Cochrane CENTRAL, and ClinicalTrials.gov were conducted until 20 December 2023, without language restrictions. **Results:** Four studies involving 644 children with premyopia aged 4–12 years were identified, with atropine concentrations ranging from 0.01% to 0.05%. The analysis focused on myopia incidence and atropine-related adverse events. Lower myopia incidence (RR, 0.62; 95% CI, 0.40–0.97 D/y; *p* = 0.03) and reduction in rapid myopia shift (≥0.5 D/1y) (RR, 0.50; 95% CI, 0.26–0.96 D/y; *p* < 0.01) were observed in the 12–24-month period. Spherical equivalent and axial length exhibited attenuated progression in the atropine group. No major adverse events were detected in either group, whereas the incidence of photophobia and allergic conjunctivitis did not vary in the 12–24-month period. **Conclusions:** Our meta-analysis supports atropine’s efficacy and safety for delaying myopia incidence and controlling progression in children with premyopia. However, further investigation is warranted due to limited studies.

## 1. Introduction

Myopia, which is a prevalent refractive error [1], has emerged as a growing global concern, with projections indicating that nearly half of the world’s population may be affected by 2050 [2], particularly in East and Southeast Asia [3]. The irreversible nature of myopia, together with its propensity to manifest early in life, underscores the urgency of addressing this issue. The onset of myopia at an early age is associated with an increased risk of development of high myopia [4,5], leading to a spectrum of ocular pathologies, including myopic maculopathy [6,7], cataracts [8], open-angle glaucoma [9], and progressive visual debilitation over time [10]. Therefore, the delay or prevention of myopia becomes imperative for the optimization of long-term visual outcomes.

Among school-age children, refraction typically undergoes gradual changes over a span of several years. As pointed out by Schmid, approximately 80% of young children exhibit hyperopia, which tends to increase until around the age of 8 years, before gradually decreasing until approximately age 20 [11]. In contrast with the traditional perception of emmetropia as being a stable state, this condition can be transient in this age group [12]. Moreover, previous studies have reported that young children with a low level of hyperopia may be at a greater risk of developing myopia later in life; individuals with hyperopia often experience slow myopic shifts, whereas individuals with myopia undergo more rapid changes [13,14]. This underscores the potential importance of preserving hyperopia for understanding and predicting the development of myopia in children.

The concept of premyopia, which was introduced by the International Myopia Institute, outlines a refractive state that is characterized by an eye power of ≤0.75 D and >−0.50 D in children. This condition is diagnosed by considering the baseline refraction, age, and other quantifiable risk factors, which collectively suggest a significant likelihood of the future development of myopia and justify preventive interventions [15]. Despite its significance, a lack of consensus remains regarding the precise definition of premyopia. In addition, the lack of standardized tools for risk stratification and management in these children complicates this scenario [16,17,18].

Among the pharmacological approaches that are available for controlling the progression of myopia, low-dose atropine has demonstrated efficacy in children with this condition in various studies [19,20,21], emerging as a main method [22]. However, the extent of its effectiveness in children with premyopia remains unclear. Recent findings from the LAMP2 trials revealed that, compared with a placebo, 0.05% atropine significantly reduced the cumulative incidence of myopia by 24.6% over a 2-year period, whereas no notable difference was observed between the 0.01% atropine and the placebo groups [18]. Conversely, another study reported that 0.01% atropine significantly prevented the myopic shift in early childhood, resulting in a 24% reduction in the incidence of myopia over 6 years compared with the placebo [17]. Moreover, several studies reported a higher rate of side effects and adverse events for topical 0.05% atropine prescribed for myopia control in children [23,24]. Therefore, the critical question regarding the effectiveness and safety of atropine in children with premyopia warrants a thorough investigation.

Therefore, this study aimed at conducting a comprehensive systematic review and meta-analysis to address this gap in the literature, focusing on the efficacy and safety of low-dose atropine for the management of children with premyopia. By meticulously reviewing the existing literature and analyzing the available data, the outcomes of this study have the potential to significantly impact the clinical decision-making and elevate the overall quality of care in the management of the progression of myopia.

## 2. Methods

### 2.1. Study Design

This meta-analysis aimed at investigating the efficacy and safety of low-dose atropine eye drops for controlling myopia in children with premyopia. This study adhered strictly to the guidelines provided by the Preferred Reporting Items for Systematic Reviews and Meta-Analyses (PRISMA) statement [25]. The methodology was pre-specified and registered on the PROSPERO website on 15 January 2024 (Registration No. PROSPERO CRD42024498463).

### 2.2. Eligibility Criteria

To ensure the validity of our analysis, we included studies that met the following criteria: (1) randomized controlled trials (RCTs) or other interventional studies; (2) studies reporting data on myopia incidence, axial length, or spherical equivalent measurements; and (3) studies involving children with premyopia, defined as an SE ranging between +1 D and −1 D, who were treated with atropine eye drops for at least 6 months.

Studies that met any of the following criteria were excluded from the analysis: (1) those including patients with myopia (SE < −1 D); (2) those that involved participants with congenital or severe ophthalmological conditions or surgical history, which may influence the outcome; and (3) those with overlapping participants.

### 2.3. Outcome Measures

The outcomes were categorized based on time, either within 1 year or over 1 year:

▪ Myopia incidence;
▪ Fast myopic shift: myopia progression exceeding 0.5 D within 1 year or at an equivalent rate;
▪ Axial length (AL);
▪ Spherical equivalent (SE);
▪ Pupil size;
▪ Accommodative amplitude;
▪ Adverse effect (photophobia, allergic conjunctivitis).

### 2.4. Data Sources and Literature Searches

Our study employed a rigorous search methodology that was carried out independently by two authors (Ssu-Hsien Lee and Bor-Yuan Tseng) across multiple databases, including PubMed, Cochrane CENTRAL, Embase, and ClinicalTrials.gov, up to 20 December 2023. We utilized a combination of keywords, such as “premyopia” and “nonmyopia”, with Medical Subject Headings to identify pertinent studies. The detailed search methods used here are outlined in Table S1. Furthermore, language restrictions were not imposed, and we meticulously reviewed reference lists, to ensure the inclusion of all relevant research.

### 2.5. Risk-of-Bias Assessment

To assess the methodological quality of the RCTs included in this analysis, we utilized the Cochrane risk-of-bias tool for randomized trials version 2 (RoB 2.0). This tool comprises five main items: the randomization process, intervention adherence, missing outcome data, outcome measurement, and selective reporting. For other study designs, we employed the Newcastle–Ottawa Scale (NOS) tool to assess potential biases; this tool consists of three domains, with up to nine stars: assessment of selection bias, comparability bias, and outcome bias.

### 2.6. Data Extraction

Two authors (Ssu-Hsien Lee and Bor-Yuan Tseng) independently performed data extraction from the selected studies. The extracted data included demographic information; study design details; atropine treatment specifics; and measurements of myopia incidence, fast myopic shift, axial length, spherical equivalent, pupil size, accommodative amplitude, adverse effects, and relevant outcomes. If essential data were absent in published articles, we contacted the corresponding authors to secure the original data.

### 2.7. Data Synthesis and Analysis

In our meta-analysis, we used a random-effects model to account for the inherent heterogeneity among the included studies via Review Manager, version 5.4. Statistical significance was set at a two-tailed *p*-value < 0.05. Effect sizes were measured based on the weighted mean difference (WMD) with 95% confidence intervals (CIs), as well as risk ratios (RRs) with 95% CIs. Heterogeneity among the studies was assessed using I^2^ statistics, using I^2^ values of 25%, 50%, and 75% as being indicative of low, moderate, and high heterogeneity, respectively. To assess the publication bias, we generated and examined funnel plots and performed Egger’s test of asymmetry.

## 3. Results

### 3.1. Literature Search

**Figure 1:**
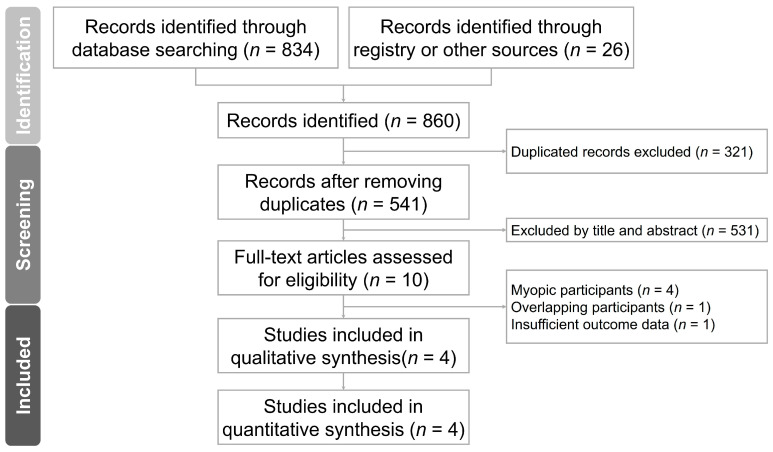
PRISMA flow chart.

The process used for our literature search and article selection is illustrated in Figure 1. Initially, 860 studies were retrieved through the database search in accordance with the PRISMA guidelines [25]. After the removal of duplicate records, a thorough examination of the titles and abstracts of the articles led to the identification of 10 studies for full-text screening. Ultimately, four studies were included in our meta-analysis. Tables S1 and S2 outline the keywords used in the search and the rationale for study exclusions, respectively. Figure S1 illustrates the funnel plot.

### 3.2. Characteristics of the Included Studies

Table 1 presents the detailed characteristics of the included studies. Our search encompassed four atropine-related studies, consisting of three RCTs and one non-RCT, involving a total of 644 children with premyopia with an average age of 7.13 ± 1.60 years. The baseline cycloplegic SE was 0.38 ± 0.44 D, and the baseline AL was 22.72 ± 0.94 mm. All studies had been conducted in Asian countries, such as India, China, and Taiwan. The atropine dosages ranged from 0.01% to 0.05%, with three studies utilizing 0.01% atropine and the others using 0.05% and 0.25% atropine. Treatment durations varied from 6 months to 2 years, with two studies lasting 2 years, one averaging 18.4 months, and only one study spanning 6 months.

Our analysis revealed consistent evidence across all four studies indicating the efficacy of low-dose atropine in children with premyopia. These findings encompassed a reduction in myopia incidence and a deceleration in the rate of myopia progression, coupled with a limitation in the elongation of AL and SE. Notably, adverse events reported across the studies primarily comprised incidences of photophobia and allergic conjunctivitis. Furthermore, investigations into secondary outcomes such as accommodation amplitude and pupil size post-atropine administration were prevalent.

Regarding the variability in atropine concentrations, the LAMP2 study delineated three distinct groups: 0.05% atropine, 0.01% atropine, and a placebo cohort. Notably, a dose-dependent effect was observed, with the 0.05% atropine group demonstrating superior efficacy in reducing myopia incidence and decelerating myopia progression compared to the 0.01% atropine group. Nevertheless, the 0.05% atropine cohort exhibited a reduction in accommodation amplitude and an increase in pupil size. However, incidences of photophobia and allergic conjunctivitis remained comparable between the two atropine concentrations.

### 3.3. Risk-of-Bias Assessment

The results of the RoB2.0 and NOS assessments are summarized in Tables S3 and S4, respectively. Most of the studies exhibited a low bias across all domains. However, in one RCT, we observed a performance bias without information about blinding, and a reporting bias because of the lack of registration of the trial. Nonetheless, the remaining studies exhibited a low risk of bias in the other domains.

### 3.4. Myopia Incidence and Fast Myopia Shift

**Figure 2:**
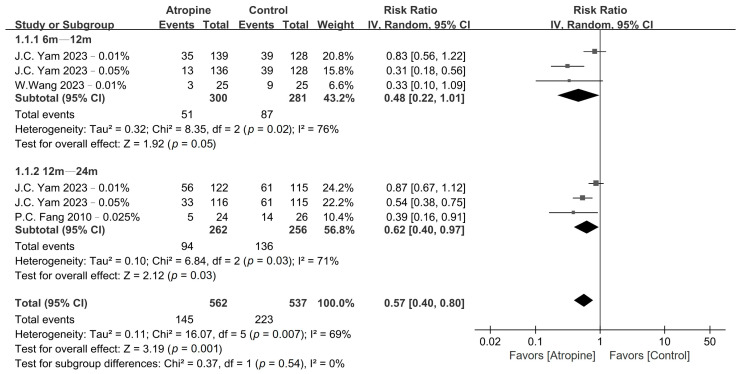
Forest plot of the risk ratio of myopia incidence between the atropine and placebo groups.

**Figure 3:**
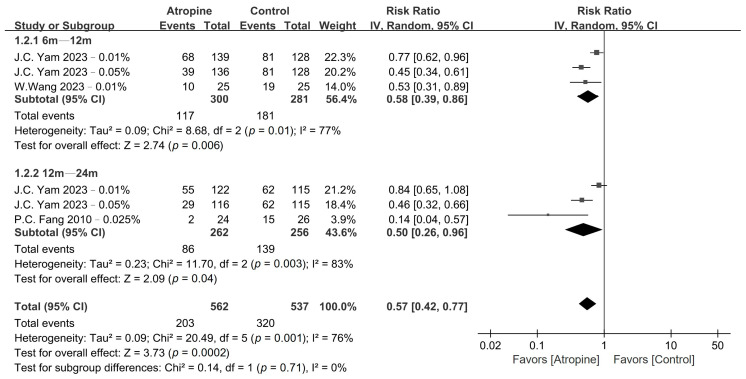
Forest plot of the risk ratio of fast myopia shift between the atropine and placebo groups.

The pooled results obtained for myopia incidence and fast myopia shift are presented in Figure 2 and Figure 3, respectively, whereas the GRADE summary of findings is provided in Table S5. Low-dose atropine exhibited a tendency to reduce myopia incidence in the 6–12-month period (RR, 0.48; 95% CI, 0.22–1.01; *p* = 0.05), with a more significant reduction over the 12–24-month period (RR, 0.62; 95% CI, 0.40–0.97; *p* = 0.03). Regarding the fast myopia shift, atropine yielded a significant reduction in this phenomenon in the 6–12-month period (RR, 0.58; 95% CI, 0.39–0.86; *p* < 0.01) and the 12–24-month period (RR, 0.50; 95% CI, 0.26–0.96; *p* = 0.04).

### 3.5. Spherical Equivalent and Axial Length

**Figure 4:**
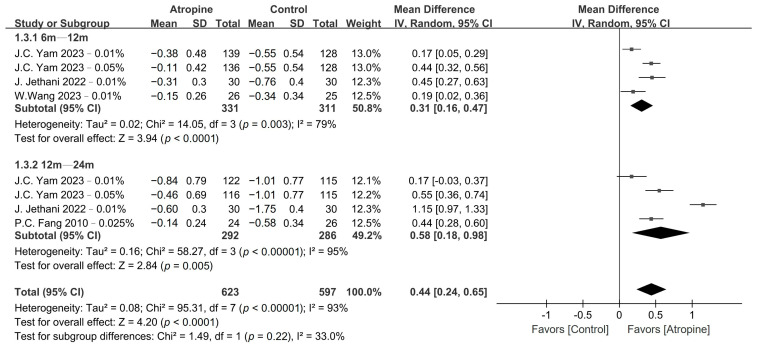
Forest plot of the mean difference in spherical equivalent between the atropine and placebo groups.

**Figure 5:**
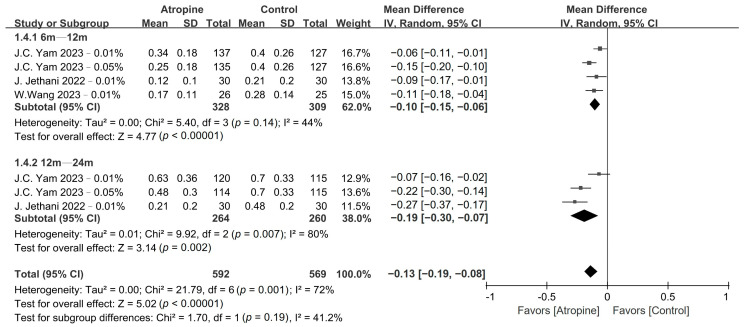
Forest plot of the mean difference in axial length between the atropine and placebo groups.

Atropine also had a significant impact on SE and AL progression, as shown in Figure 4 and Figure 5, respectively. Low-dose atropine resulted in a significantly slower SE progression vs. the placebo in the 6–12-month period (WMD, 0.31 D; 95% CI, 0.16–0.47 D; *p* < 0.01), and an even more significant slowing of SE progression over the 12–24-month period (WMD, 0.58 D; 95% CI, 0.18–0.98 D; *p* < 0.01). The axial length also exhibited a similar trend, with AL progression being significantly slower in the atropine group in the 6–12-month period (WMD, −0.10 mm; 95% CI, −0.15 to −0.06 mm; *p* < 0.01) and even more so over the 12–24-month period (WMD, −0.19 mm; 95% CI, −0.30 to −0.07 mm; *p* < 0.01).

### 3.6. Adverse Events

**Figure 6:**
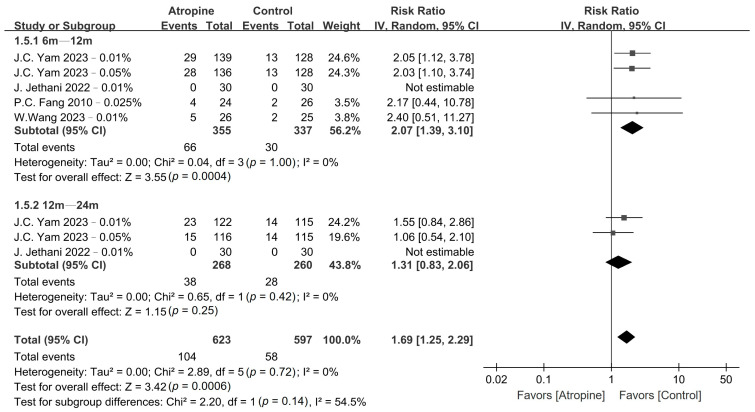
Forest plot of the risk ratio of photophobia incidence between the atropine and placebo groups.

**Figure 7:**
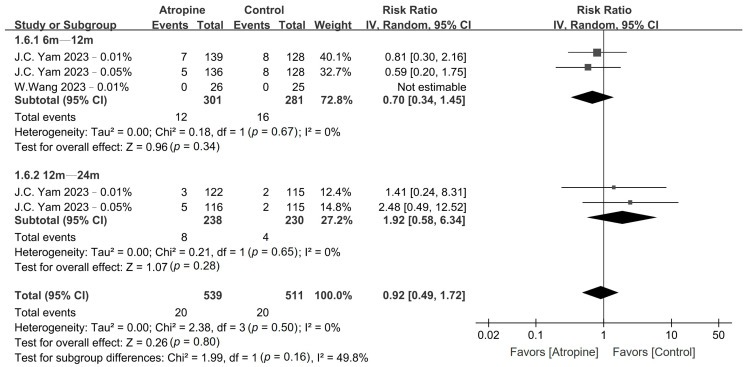
Forest plot of the risk ratio of allergic conjunctivitis incidence between the atropine and placebo groups.

No major adverse events were observed in either the atropine or the placebo group. However, the patients in the atropine group exhibited a significantly higher incidence of photophobia compared with those in the placebo group in the 6–12-month period (RR, 2.07; 95% CI, 1.39–3.10; *p* < 0.01); however, no such difference was observed over the 12–24-month period (RR, 1.31; 95% CI, 0.83–2.06; *p* = 0.25), as shown in Figure 6. Conversely, there was no difference in the incidence of allergic conjunctivitis between the atropine and placebo groups in the 6–12-month period (RR, 0.70; 95% CI, 0.34–1.45; *p* = 0.34) or the 12–24-month period (RR, 1.92; 95% CI, 0.58–6.34; *p* = 0.28), as depicted in Figure 7.

### 3.7. Accommodation Amplitude and Pupil Size

**Figure 8:**
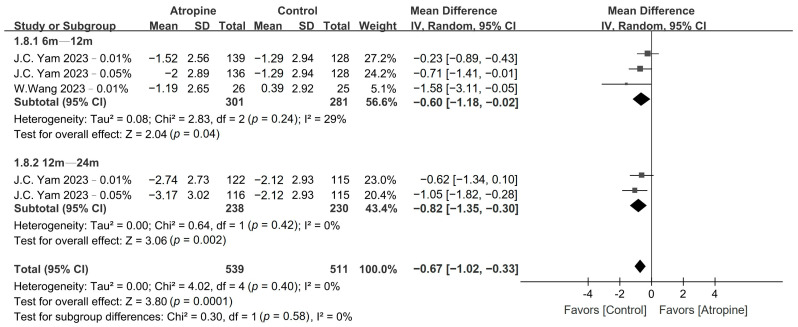
Forest plot of the mean difference in accommodation amplitude incidence between the atropine and placebo groups.

**Figure 9:**
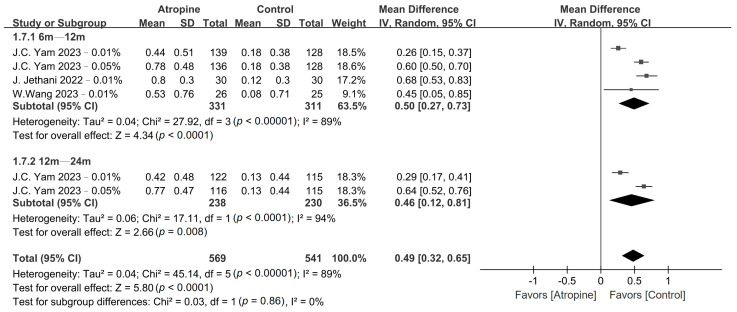
Forest plot of the mean difference in pupil size incidence between the atropine and placebo groups.

The patients who were treated with low-dose atropine exhibited a significantly lower accommodation amplitude compared with the placebo group in the 6–12-month period (WMD, −0.60 D; 95% CI, −1.18 to −0.02 D; *p* = 0.04), and even more so over the 12–24-month period (WMD, −0.82 D; 95% CI, −1.35 to −0.30 D; *p* < 0.01), as shown in Figure 8. Moreover, children in the atropine group also exhibited a larger pupil size than did those in the placebo group in the 6–12-month period (WMD, 0.50 mm; 95% CI, 0.27–0.73 mm; *p* < 0.01) and in the 12–24-month period (WMD, 0.46 mm; 95% CI, 0.12–0.81 mm; *p* < 0.01), as depicted in Figure 9.

## 4. Discussion

To the best of our knowledge, this study represents a pioneering systematic review and meta-analysis that evaluated the efficacy and safety of low-dose atropine in children with premyopia. Our findings suggest that low-dose atropine is effective in reducing the incidence of myopia, slowing down the shift toward fast myopia, and curbing the progression of SE and AL. These results agree with observations performed in children with myopia [24,27,28]. Moreover, there were no discernible differences in the incidence of photophobia and allergic conjunctivitis over a period exceeding 1 year. Conversely, although children in the atropine group exhibited a lower accommodation amplitude and a larger pupil size, these differences may not be clinically significant. Overall, the results of our study suggest that low-dose atropine is both effective and safe, even in children without pre-existing myopia. Nevertheless, due to the limited number of studies, some confidence intervals of RR are close to 1, warranting further investigation to validate our findings and provide more robust evidence.

Premyopia, alternatively termed low hyperopia reserve [12], is associated with various factors, including parental and environmental myopiogenic effects [29]. The definitions of premyopia are highly divergent, with some researchers describing it as a child experiencing a change in glass power greater than 0.5 D per year on the myopic side and a spherical equivalent lower than +1.00 D [30]. Various studies, such as those reported by Fang et al. [16] and Wang et al. [17], employed distinct SE criteria for premyopia, underscoring the need for standardized criteria. Larger research studies, such as LAMP2 [18] and ATOM3 [31], have contributed to this ongoing debate. The LAMP2 study defined premyopia as an SE of +1–0 D in 4–9-year-old children. In addition, the design of the ATOM3 study included patients with a family history of myopia presenting with low hyperopia or low myopia, encompassing 5–9-year-old children with an SE range of +1.00 to −1.50 D. The ongoing debate regarding the definition of premyopia necessitates further exploration, to identify genuine factors associated with this condition and distinguish young individuals with premyopia from their peers with stable emmetropia.

Our research underscores the substantial efficacy of low-dose atropine in reducing myopia incidence and fast myopia shift, as well as diminishing SE and AL progression in children with premyopia. The significance of delaying the onset of myopia is paramount in mitigating the development of high myopia [32,33], thus emphasizing the importance of premyopia control, which may prove to be more effective than interventions in individuals who already have myopia. The varying efficacy of low-dose atropine observed in patients with myopia and premyopia may be attributed to the differing rates of SE and AL progression recorded over time between these two conditions. Previous research indicates that children with premyopia undergo more rapid changes in refraction and axial length, with the pace gradually slowing down after the onset of myopia [34,35]. Nevertheless, the mechanism underlying the observation that children with premyopia with rapid AL and SE progression exhibited a better response to low-dose atropine remains unclear. In the realm of myopia research, an association is often established between this condition and choroidal thickness [36,37]. The possible underlying mechanisms include an atropine-induced choroidal thickening through the stimulation of dopamine release [38]. In addition, atropine may mediate choroidal blood vessel relaxation, potentially increasing capillary permeability, leading to choroidal thickening [39]. We hypothesized that, in individuals with premyopia, the mechanism of action of atropine is similar to that observed in patients with myopia. Nevertheless, beyond lifestyle modifications, atropine emerges as a pivotal method for controlling myopia, especially in patients with identifiable myopia risks [40].

Because of the limited number of available studies, in this study, we were unable to conduct subgroup analyses regarding the concentration of atropine. However, the efficacy of atropine for myopia control exhibited a dose-dependent relationship, with higher concentrations enhancing effectiveness but also resulting in more severe adverse events [41]. The debate on the optimal concentration of atropine for children with myopia persists. Notably, although 0.01% atropine has proven to be effective in slowing myopia progression down in Asian children [41], its efficacy has not been replicated in American children compared with a placebo [42]. Moreover, the LAMP study designates 0.05% atropine as the optimal concentration, striking a balance between efficacy and adverse events [41]. However, the confirmation of the optimal concentration for use in children with premyopia requires further research.

In terms of adverse events, our analysis revealed a higher incidence of photophobia in the atropine group during the 6–12-month period, with no significant differences in allergic conjunctivitis and photophobia detected between the groups in the 12–24-month period. Consistent with the existing myopia studies, photophobia and blurred near vision are adverse effects that are commonly reported to be associated with atropine therapy [24,32]. Despite the initial adverse effects related to atropine usage, the prolonged use of this medication appears to mitigate these effects [43]. This mitigation is likely caused by drug-tolerance and compensation mechanisms. Our analysis further demonstrated that the atropine group exhibited a lower accommodation amplitude and a larger pupil size; however, these findings appeared to be subclinical. More specifically, low-dose atropine resulted in a loss of less than 1 D of accommodation and an increase of about 0.5 mm in pupil size. However, these mild side effects were deemed to be clinically insignificant, as observed in studies such as ATOM2, in which no change or loss in distance or near visual acuity was observed. Notably, pupil size and accommodation returned to the baseline levels at approximately 2 months after the discontinuation of atropine [21]. Consequently, in children with myopia, as these changes are subclinical and reversible, adherence to low-dose atropine drop therapy is generally good. Previous reports indicate that over 90% of children adhere to the treatment more than six times per week [44]. Similarly, we infer that, due to the low impact on the quality of life, there might be high adherence to low-dose atropine in premyopic children.

Beyond atropine intervention, various strategies are employed to impede the progression to myopia among children with premyopia. A noteworthy approach involves the repeated application of low-level red light, utilizing a device emitting 650 nm visible red light. When administered twice daily in sessions of 3 min, this novel therapy has demonstrated efficacy in children with myopia [45,46], and recent studies have corroborated its effectiveness in children with premyopia [47,48]. The 12-month incidence of myopia was 40.8% in the intervention group and 61.3% in the control group, whereas SE and AL progression was also reduced in the intervention group [48]. However, its susceptibility to a rebound effect is a notable drawback [49]. Another viable method consists of increasing the time spent outdoors [50,51], particularly under robust sunlight exposure [52,53], which has proven to be effective in controlling myopia progression both in children with myopia and in those with premyopia. Furthermore, family health education serves as an additional avenue for myopia control [54,55,56]. In contrast to pharmacological approaches, there is a paucity of optical methods, such as orthokeratology, for the cohort with premyopia. Additional research is imperative to ascertain the effectiveness of these methodologies and explore potential synergies arising from their combination.

## 5. Limitations

Our study had several limitations. Firstly, the diverse definitions of myopia and premyopia used across the studies included in the present analysis may have introduced variability in our results. Fortunately, the fast myopia shift, which remains unaffected by the definitions, exhibited a similar trend to that observed for myopia incidence, supporting the robustness of our findings. Secondly, the limited number of studies available may have impacted the statistical power and hindered our ability to conduct subgroup analyses, such as exploring the effects of different atropine concentrations and treatment durations. Therefore, further investigations are imperative to determine the optimal atropine concentration and treatment duration for children with premyopia. Thirdly, the rebound effect of atropine was not assessed because of the scarcity of relevant studies. Future research should focus on designing studies specifically addressing the rebound effect of atropine in children with premyopia. Finally, the fact that all the studies included here were conducted in Asia may restrict the generalizability of our findings to populations with diverse genetic, environmental, or lifestyle factors influencing myopia progression. Therefore, it is essential to conduct further studies involving diverse ethnic groups to enhance the external validity of our results.

## 6. Conclusions

Our meta-analysis highlighted the effectiveness of low-dose atropine in controlling myopia among children with premyopia, as evidenced by a decrease in myopia incidence and fast myopia shift, together with a notable reduction in SE and AL progression. In terms of safety, our findings revealed the absence of major adverse events, and the minor differences in adverse events observed between the atropine and placebo groups lacked clinical significance. In summary, our findings support the safety and efficacy of low-dose atropine for individuals with premyopia. Nevertheless, due to the current limitations in evidence, further research is imperative to determine the optimal dosage and treatment duration, for the establishment of more conclusive recommendations.

## Figures and Tables

**Table 1:** Characteristics of the included studies.

Study	Study Design	Atropine %, Treatment Time	*n*	Age (years)	Baseline SE (D)	Baseline AL (mm)	Country
J.C. Yam 2023 [18]	RCT	0.05%, 2 y	160	6.86 ± 1.42	0.50 ± 0.33	22.82 ± 0.72	Hong Kong
		0.01%, 2 y	159	6.88 ± 1.35	0.51 ± 0.33	22.89 ± 0.70	
		Placebo, 2 y	155	6.75 ± 1.27	0.53 ± 0.31	22.80 ± 0.64	
W. Wang 2023 [17]	RCT	0.01%, 6 m	30	8.60 ± 1.72	−0.19 ± 0.28	23.59 ± 0.77	China
		Placebo, 6 m	30	8.50 ± 1.74	−0.21 ± 0.32	23.61 ± 0.75	
J. Jethani 2022 [26]	RCT	0.01%, 2 y	30	7.70 ± 2.10	N/A	20.80 ± 0.60	India
		Placebo, 2 y	30	7.20 ± 1.90		21.00 ± 0.50	
P.C. Fang 2010 [16]	Non-RCT	0.025%, 18.4 m *	24	7.60 ± 1.70	−0.31 ± 0.45	N/A	Taiwan
		Placebo, 16.3 m *	26	8.20 ± 2.10	−0.17 ± 0.50		

Note: m: months; y: years; N/A: not available. All data are reported as the mean ± SD, if not marked otherwise. * Average follow-up time.

## Data Availability

Data are contained within the article or Supplementary Materials. Further inquiries can be directed to the corresponding author.

## Supplementary Materials

The following are available online at https://www.mdpi.com/article/10.3390/jcm13051506/s1, Table S1: keywords and search results in different databases, Table S2: excluded studies and reasons [57,58,59,60,61,62], Table S3: detailed quality assessment of included studies using Cochrane risk-of-bias 2 tool (RoB 2.0) [17,18,26], Table S4: detailed quality assessment of included studies using Newcastle–Ottawa Scale (NOS) [16], Table S5: GRADE summary of findings, Table S6: PRISMA checklist, Figure S1: funnel plot of myopia incidence.
