# Supplementary material for: Efficacy and Safety of Low-Dose Atropine on Myopia Prevention in Premyopic Children: Systematic Review and Meta-Analysis
Source: J Clin Med. 2024 Mar 5;13(5):1506. doi: 10.3390/jcm13051506 (PMC10932201; doi:10.3390/jcm13051506)
Supplement: Supplementary file 1 [file jcm-13-01506-s001.zip › jcm-2851529-supplementary.pdf]

*Supplementary Material*

# Efficacy and Safety of Low-dose Atropine for Myopia control in Premyopic children: Systematic Review and Meta-Analysis

**Ssu-Hsien Lee [1,†], Bor-Yuan Tseng [1,†], Jen-Hung Wang [2], Cheng-Jen Chiu [3,4,*]**

[1] School of Medicine, Tzu Chi University, Hualien 970, Taiwan
[2] Department of Medical Research, Buddhist Tzu Chi General Hospital, Hualien 970, Taiwan
[3] Department of Ophthalmology and Visual Science, Tzu Chi University, Hualien 970, Taiwan
[4] Department of Ophthalmology, Hualien Tzu Chi Hospital, the Buddhist Tzu Chi Medical Foundation, Hualien 970, Taiwan
[†] These authors contributed equally to this work.
* Correspondence: drcjchiu@tzuchi.com.tw

# Supplement

## Table S1. Keywords and search results in different databases

| Database | Keyword | Filter | Date | Results |
|---|---|---|---|---|
| PubMed | (premyopia OR premyopic OR non-myopic OR nonmyopic OR non-myopia OR nonmyopia) AND myopia | NA | December 03, 2023 | 387 |
| Embase | (premyopia.mp. OR premyopic.mp. OR non-myopic.mp. OR nonmyopic.mp. OR non-myopia.mp. OR nonmyopia.mp.) AND (myopia.mp. or myopia/) | NA | December 03, 2023 | 409 |
| Cochrane CENTRAL | (premyopia OR premyopic OR non-myopic OR nonmyopic OR non-myopia OR nonmyopia) AND (myopia or myopia [MeSH]) | NA | December 03, 2023 | 38 |
| ClinicalTrials.gov | (premyopia OR premyopic OR non-myopic OR nonmyopic OR non-myopia OR nonmyopia) AND myopia | Condition or disease | December 03, 2023 | 24 |

NA: not applied

**Table S2. Excluded studies and reasons**

| Study | Reason for exclusion |
|---|---|
| Effects of 0.01% atropine eye drops on the prevention of myopia onset among schoolchildren: a randomized, double-blind, controlled trial [57] | Overlapping participants |
| Prevention of myopia onset and progression with 0.01% atropine solution among school-age children [58] | Myopic participants |
| The increasing prevalence of myopia in junior high school students in the Haidian District of Beijing, China: a 10-year population-based survey [59] | Insufficient outcome data |
| Longitudinal changes in corneal curvature and its relationship to axial length in the Correction of Myopia Evaluation Trial (COMET) cohort [60] | Myopic participants |
| Eye growth pattern of myopic children wearing spectacle lenses with aspherical lenslets compared with non-myopic children [61] | Myopic participants |
| Characteristics of responders to atropine 0.01% as treatment in Asian myopic children [62] | Myopic participants |

**Table S3. Detailed quality assessment of included studies using Cochrane risk of bias 2 tool (RoB 2.0)**

| First Author | Year | Randomization process | Intervention adherence | Missing outcome data | Outcome measurement | Selective reporting | Overall |
|---|---|---|---|---|---|---|---|
| J. Jethani **[26]** | 2022 | L | S | L | L | S | S |
| J.C. Yam **[18]** | 2023 | L | L | L | L | L | L |
| W. Wang **[17]** | 2023 | L | L | L | L | L | L |

H, high risk of bias; L, low risk of bias; S, some concern of risk of bias.

**Table S4. Detailed quality assessment of included studies using Newcastle-Ottawa Scale (NOS)**

| First Author | Year | Selection | Comparability | Outcome | Overall |
|---|---|---|---|---|---|
| P.C. Fang **[16]** | 2010 | 4/4 | 2/2 | 3/3 | 9/9 |

## Table S5. GRADE Summary of Findings Table

| Effect | | | | Certainty |
|---|---|---|---|---|
| **Atropine** | **Placebo** | **Relative (95% CI)** | **Absolute (95% CI)** | |
| **Myopia Incidence - 6m—12m** | | | | |
| 51/300 (17.0%) | 87/281 (31.0%) | **RR 0.48** (0.22 to 1.01) | **161 fewer per 1,000** (from 241 fewer to 3 more) | ⊕⊕⊕◯ Moderate |
| **Myopia Incidence - 12m—24m** | | | | |
| 94/262 (36.3%) | 136/256 (53.1%) | **RR 0.62** (0.42 to 0.97) | **202 fewer per 1,000** (from 319 fewer to 16 fewer) | ⊕◯◯◯ Very low |
| **Fast Myopia Shift - 6m—12m** | | | | |
| 117/300 (39.0%) | 181/281 (64.4%) | **RR 0.58** (0.39 to 0.86) | **271 fewer per 1,000** (from 393 fewer to 90 fewer) | ⊕⊕⊕◯ Moderate |
| **Fast Myopia Shift - 12m—24m** | | | | |
| 86/262 (32.8%) | 139/256 (54.3%) | **RR 0.50** (0.26 to 0.96) | **271 fewer per 1,000** (from 402 fewer to 22 fewer) | ⊕◯◯◯ Very low |
| **Spherical Equivalent - 6m—12m** | | | | |
| 331 | 311 | - | MD **0.31 higher** (0.16 higher to 0.47 higher) | ⊕⊕◯◯ Low |

| Effect | | | | Certainty |
|---|---|---|---|---|
| **Atropine** | **Placebo** | **Relative (95% CI)** | **Absolute (95% CI)** | |
| **Spherical Equivalent - 12m—24m** | | | | |
| 292 | 286 | - | MD **0.58 higher** (0.18 higher to 0.98 higher) | ⊕◯◯◯ Very low |
| **Axial Length - 6m—12m** | | | | |
| 328 | 309 | - | MD **0.1 lower** (0.15 lower to 0.06 lower) | ⊕⊕◯◯ Low |
| **Axial Length - 12m—24m** | | | | |
| 264 | 260 | - | MD **0.19 lower** (0.3 lower to 0.07 lower) | ⊕◯◯◯ Very low |
| **Photophobia Incidence - 6m—12m** | | | | |
| 66/355 (18.6%) | 30/337 (8.9%) | **RR 2.07** (1.39 to 3.10) | **95 more per 1,000** (from 35 more to 187 more) | ⊕◯◯◯ Very low |
| **Photophobia Incidence - 12m—24m** | | | | |
| 38/268 (14.2%) | 28/260 (10.8%) | **RR 1.31** (0.83 to 2.06) | **33 more per 1,000** (from 18 fewer to 114 more) | ⊕⊕◯◯ Low |

| Effect | | | | Certainty |
|---|---|---|---|---|
| Atropine | Placebo | Relative (95% CI) | Absolute (95% CI) | |
| **Allergic Conjunctivitis Incidence - 6m—12m** | | | | |
| 12/301 (4.0%) | 16/281 (5.7%) | **RR 0.70** (0.34 to 1.45) | **17 fewer per 1,000** (from 38 fewer to 26 more) | ⊕⊕◯◯ Low |
| **Allergic Conjunctivitis Incidence - 12m—24m** | | | | |
| 8/238 (3.4%) | 4/230 (1.7%) | **RR 1.92** (0.58 to 6.34) | **16 more per 1,000** (from 7 fewer to 93 more) | ⊕⊕◯◯ Low |
| **Pupil Size - 6m—12m** | | | | |
| 331 | 311 | - | MD **0.5 higher** (0.27 higher to 0.73 higher) | ⊕⊕◯◯ Low |
| **Pupil Size - 12m—24m** | | | | |
| 238 | 230 | - | MD **0.46 higher** (0.12 higher to 0.81 higher) | ⊕⊕⊕◯ Moderate |
| **Accommodation Amplitude - 6m—12m** | | | | |
| 301 | 281 | - | MD **0.6 lower** (1.18 lower to 0.02 lower) | ⊕⊕⊕◯ Moderate |

| Effect | | | | Certainty |
|---|---|---|---|---|
| Atropine | Placebo | Relative (95% CI) | Absolute (95% CI) | |
| **Accommodation Amplitude - 12m—24m** | | | | |
| 238 | 230 | - | MD **0.82 lower** (1.35 lower to 0.3 lower) | ⊕⊕⊕◯ Moderate |

## Table S6. PRISMA Checklist

| Section and Topic | # | Checklist item | Location |
|---|---|---|---|
| **TITLE** | | | |
| Title | 1 | Identify the report as a systematic review. | Title |
| **ABSTRACT** | | | |
| Abstract | 2 | See the PRISMA 2020 for Abstracts checklist. | Method |
| **INTRODUCTION** | | | |
| Rationale | 3 | Describe the rationale for the review in the context of existing knowledge. | Introduction |
| Objectives | 4 | Provide an explicit statement of the objective(s) or question(s) the review addresses. | Introduction |
| **METHODS** | | | |
| Eligibility criteria | 5 | Specify the inclusion and exclusion criteria for the review and how studies were grouped for the syntheses. | Methods |
| Information sources | 6 | Specify all databases, registers, websites, organisations, reference lists and other sources searched or consulted to identify studies. Specify the date when each source was last searched or consulted. | Methods |
| Search strategy | 7 | Present the full search strategies for all databases, registers and websites, including any filters and limits used. | Methods, Table S1 |
| Selection process | 8 | Specify the methods used to decide whether a study met the inclusion criteria of the review, including how many reviewers screened each record and each report retrieved, whether they worked independently, and if applicable, details of automation tools used in the process. | Methods |
| Data collection process | 9 | Specify the methods used to collect data from reports, including how many reviewers collected data from each report, whether they worked independently, any processes for obtaining or confirming data from study investigators, and if applicable, details of automation tools used in the process. | Methods |
| Data items | 10a | List and define all outcomes for which data were sought. Specify whether all results that were compatible with each outcome domain in each study were sought (e.g., for all measures, time points, analyses), and if not, the methods used to decide which results to collect. | Methods |

| | | | |
|---|---|---|---|
| | 10b | List and define all other variables for which data were sought (e.g., participant and intervention characteristics, funding sources). Describe any assumptions made about any missing or unclear information. | Methods Table 1 |
| Study risk of bias assessment | 11 | Specify the methods used to assess risk of bias in the included studies, including details of the tool(s) used, how many reviewers assessed each study and whether they worked independently, and if applicable, details of automation tools used in the process. | Methods, TablesS3, S4 |
| Effect measures | 12 | Specify for each outcome the effect measure(s) (e.g., risk ratio, mean difference) used in the synthesis or presentation of results. | Methods |
| Synthesis methods | 13a | Describe the processes used to decide which studies were eligible for each synthesis (e.g., tabulating the study intervention characteristics and comparing against the planned groups for each synthesis (item #5)). | Methods, Figure 1, |
| | 13b | Describe any methods required to prepare the data for presentation or synthesis, such as handling of missing summary statistics, or data conversions. | Methods |
| | 13c | Describe any methods used to tabulate or visually display results of individual studies and syntheses. | Methods |
| | 13d | Describe any methods used to synthesize results and provide a rationale for the choice(s). If meta-analysis was performed, describe the model(s), method(s) to identify the presence and extent of statistical heterogeneity, and software package(s) used. | Methods |
| | 13e | Describe any methods used to explore possible causes of heterogeneity among study results (e.g., subgroup analysis, meta-regression). | Methods |
| | 13f | Describe any sensitivity analyses conducted to assess robustness of the synthesized results. | Methods |
| Reporting bias assessment | 14 | Describe any methods used to assess risk of bias due to missing results in a synthesis (arising from reporting biases). | Methods, Tables S3, S4 |
| Certainty assessment | 15 | Describe any methods used to assess certainty (or confidence) in the body of evidence for an outcome. | Methods, Table S5 |
| **RESULTS** | | | |
| Study selection | 16a | Describe the results of the search and selection process, from the number of records identified in the search to the number of studies included in the review, ideally using a flow diagram. | Results, Figure 1 |

| | | | |
|---|---|---|---|
| | 16b | Cite studies that might appear to meet the inclusion criteria, but which were excluded, and explain why they were excluded. | Results, Table S2 |
| Study characteristics | 17 | Cite each included study and present its characteristics. | Results, Table 1 |
| Risk of bias | 18 | Present assessments of risk of bias for each included study. | Results, Tables S3, S4 |
| Results of individual studies | 19 | For all outcomes, present, for each study: (a) summary statistics for each group (where appropriate) and (b) an effect estimates and its precision (e.g., confidence/credible interval), ideally using structured tables or plots. | Figures 2-9 |
| Results of syntheses | 20a | For each synthesis, briefly summarise the characteristics and risk of bias among contributing studies. | Results, Tables S3, S4 |
| | 20b | Present results of all statistical syntheses conducted. If meta-analysis was done, present for each the summary estimate and its precision (e.g., confidence/credible interval) and measures of statistical heterogeneity. If comparing groups, describe the direction of the effect. | Results, Figures 2-9 |
| | 20c | Present results of all investigations of possible causes of heterogeneity among study results. | Results, Figures 2-9 |
| | 20d | Present results of all sensitivity analyses conducted to assess the robustness of the synthesized results. | Not applicable |
| Reporting biases | 21 | Present assessments of risk of bias due to missing results (arising from reporting biases) for each synthesis assessed. | Results, Figures S1 |
| Certainty of evidence | 22 | Present assessments of certainty (or confidence) in the body of evidence for each outcome assessed. | Table S5 |
| **DISCUSSION** | | | |
| Discussion | 23a | Provide a general interpretation of the results in the context of other evidence. | Discussion |
| | 23b | Discuss any limitations of the evidence included in the review. | Discussion |
| | 23c | Discuss any limitations of the review processes used. | Discussion |
| | 23d | Discuss implications of the results for practice, policy, and future research. | Discussion |

| OTHER INFORMATION | | | |
|---|---|---|---|
| Registration and protocol | 24a | Provide registration information for the review, including register name and registration number, or state that the review was not registered. | Methods |
| | 24b | Indicate where the review protocol can be accessed, or state that a protocol was not prepared. | Methods |
| | 24c | Describe and explain any amendments to information provided at registration or in the protocol. | Methods |
| Support | 25 | Describe sources of financial or non-financial support for the review, and the role of the funders or sponsors in the review. | Funding |
| Competing interests | 26 | Declare any competing interests of review authors. | Conflicts of Interest |
| Availability of data, code and other materials | 27 | Report which of the following are publicly available and where they can be found: template data collection forms; data extracted from included studies; data used for all analyses; analytic code; any other materials used in the review. | Results, Table 1, Tables S1-S5 |

**Figure S1. Funnel Plot of Myopia Incidence**

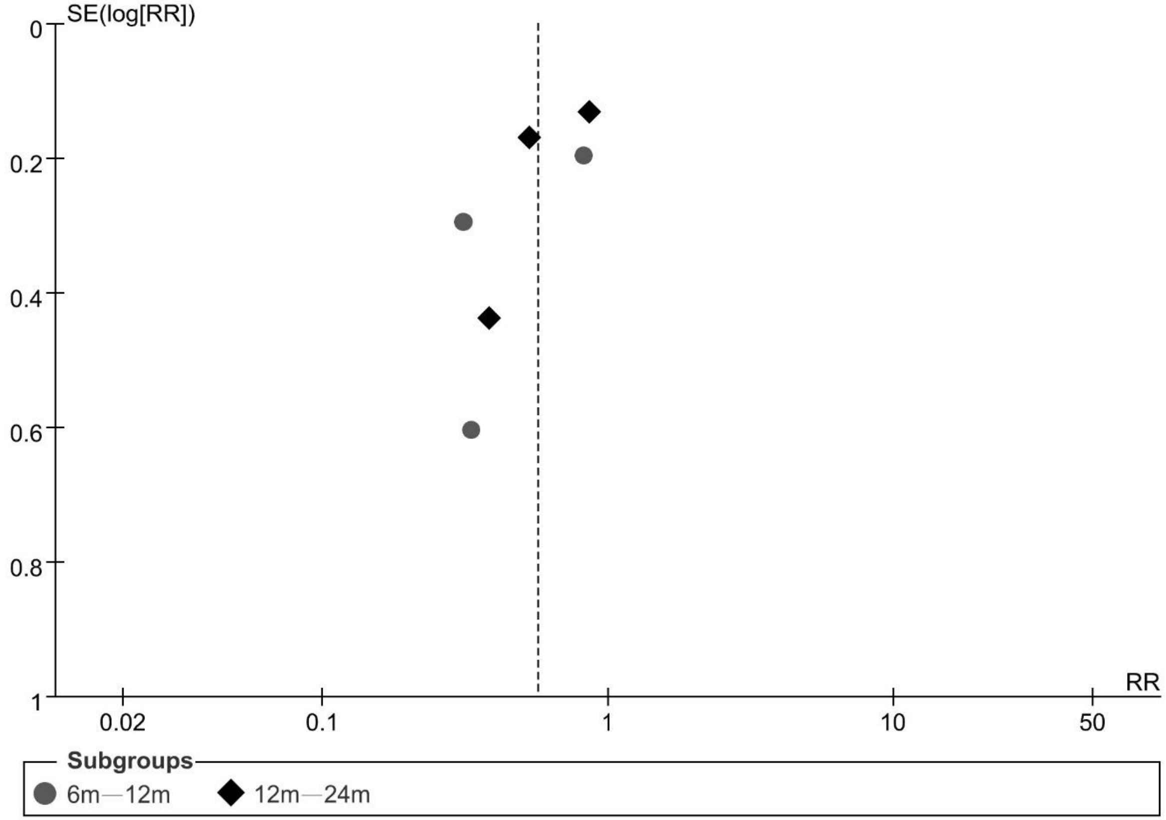

## References


[16] Fang PC, Chung MY, Yu HJ, Wu PC. Prevention of myopia onset with 0.025% atropine in premyopic children. *J Ocul Pharmacol Ther.* Aug 2010;26(4):341-345

[17] Wang W, Zhang F, Yu S, et al. Prevention of myopia shift and myopia onset using 0.01% atropine in premyopic children - a prospective, randomized, double-masked, and crossover trial. *Eur J Pediatr.* Jun 2023;182(6):2597-2606

[18] Yam JC, Zhang XJ, Zhang Y, et al. Effect of Low-Concentration Atropine Eyedrops vs Placebo on Myopia Incidence in Children: The LAMP2 Randomized Clinical Trial. *Jama.* Feb 14 2023;329(6):472-481

[26] Jethani J. Efficacy of low-concentration atropine (0.01%) eye drops for prevention of axial myopic progression in premyopes. *Indian J Ophthalmol.* Jan 2022;70(1):238-240

[57] Shiao Y, Yong L, Weiqun W, et al. Effects of 0.01% atropine eye drops on the prevention of myopia onset among schoolchildren: a randomized, double-blind, controlled trial. *Chinese Journal of Experimental Ophthalmology.* 2022:533-540.

[58] Jingyi L, Furong H, Xiaowei Z, Changan L, Xiaojie H. Prevention of myopia onset and progression with 0.01% atropine solution among school-age children. *Chinese Journal of School Health* 2018;39(3):432-435.

[59] Li Y, Liu J, Qi P. The increasing prevalence of myopia in junior high school students in the Haidian District of Beijing, China: a 10-year population-based survey. *BMC Ophthalmol.* Jun 12 2017;17(1):88.

[60] Scheiman M, Gwiazda J, Zhang Q, et al. Longitudinal changes in corneal curvature and its relationship to axial length in the Correction of Myopia Evaluation Trial (COMET) cohort. *J Optom.* Jan-Mar 2016;9(1):13-21.

[61] Wong YL, Li X, Huang Y, et al. Eye growth pattern of myopic children wearing spectacle lenses with aspherical lenslets compared with non-myopic children. *Ophthalmic Physiol Opt.* Sep 15 2023.

[62] Lee LC, Hsieh MW, Chen YH, Chen PL, Chien KH. Characteristics of responders to atropine 0.01% as treatment in Asian myopic children. *Sci Rep.* May 5 2022;12(1):7380.
